# A New Overview of Sex Bias in Fungal Infections

**DOI:** 10.3390/jof10090607

**Published:** 2024-08-26

**Authors:** Hari H. Rao, Erin E. McClelland

**Affiliations:** Biomedical Sciences Division, Marian University College of Osteopathic Medicine, Indianapolis, IN 46222, USA; hrao229@marian.edu

**Keywords:** fungal infections/colonizations, sex susceptibility, biological sex, microbiome

## Abstract

Fungal infections often disproportionately affect males over females. Since the NIH mandated in 2016 that researchers test their hypotheses in both biological sexes, numerous other fungal infections/colonizations have been found to exhibit sex-specific patterns. These patterns have been observed in various species, including mice, drosophila, cats, and bats, suggesting significant implications for understanding these diseases and developing treatments. Despite the recognition of this sex bias, primary research explaining its underlying causes or mechanisms remains limited. Current evidence suggests that potential causes might be linked to sex hormones, genetic expression, and evolutionary behaviors. This review consolidates recent data on sex bias in fungal infections or colonizations among different species and proposes future research directions to address existing gaps. Thus, this review advances the comprehension of the intricate relationships between biological sex, fungal infections, and broader health implications.

## 1. Introduction

Fungi are a distinct class of microorganisms, most of which are free-living in nature where they function as decomposers in the energy cycle and are classified into nine different phyla: Opisthosporidia, Chytridiomycota, Neocallimastigomycota, Blastocladiomycota, Zoopagomycota, Mucoromycota, Glomeromycota, Basidiomycota and Ascomycota [1]. Of the more than 200,000 known species, fewer than 100 have been reported to produce disease in humans [2]. Fungal infections may be classified into four categories based on their infection site: superficial, cutaneous, subcutaneous, and deep. Superficial mycoses are limited to the stratum corneum of the epidermis. In contrast, cutaneous mycoses extend to deeper epidermal layers and involve the integument and its appendages, such as hair and nails. Subcutaneous mycoses affect subcutaneous tissues, typically following traumatic inoculation. Deep mycoses, the most severe, impact internal organs and the central nervous system, posing significant health risks [3].

Worldwide, there are over 80 million severe cases of fungal infections annually [4], resulting in approximately 3.8 million deaths per year [5]. Unlike bacterial and viral diseases, fungal infections seldom cause acute illness. Nevertheless, the incidence of these infections continues to increase annually due to several factors. One contributing factor is the expanding population that is susceptible to fungal infections. Advancements in modern medicine have prolonged longevity, thereby increasing the elderly demographic, who are particularly susceptible to these infections. Furthermore, other advancements, such as successful organ transplants and immunotherapies for cancer treatment, have resulted in a growing population of immunocompromised individuals, further exacerbating patient susceptibility to fungal infections [6].

In addition, current methods to treat fungal infections are limited. The long-term use of antifungal drugs in agriculture is leading to an increase in drug-resistant fungi, such as the rise in cases of azole-resistant *Aspergillus fumigatus* [7,8]. With the advent of warmer average global temperatures, the risk of fungal infections typically limited to tropical environments is expanding to areas that are more temperate, and there is a concern that these fungi may mutate and acquire the ability to infect mammals [9]. Additionally, with global warming comes the occurrence of more unusual and stronger weather patterns (i.e., floods, storms, and hurricanes), which disperse and aerosolize fungi and their spores, resulting in increasing numbers of infections [10].

Various soil-borne fungal pathogens, including *Talaromyces marneffei, Blastomyces, Histoplasma*, and *Paracoccidioides*, have heightened frequency or expanded geographical range due to climate-induced disruptions [10]. For example, cases of coccidioidomycosis, historically considered endemic to the southwestern United States, have now been observed in Nebraska and as far north as Washington state [11]. Given the significant health risks posed by fungal infections and the ongoing increase in reported cases, it is imperative to recognize the disproportionate impact of these infections on specific segments of the population.

In previous reviews, we examined sex bias in *Cryptococcus neoformans* (*C. neoformans*), *Candida albicans* (*C. albicans*), and *Paracoccidioides brasiliensis* (*P. brasiliensis*) infections [12,13,14]. In 2016, the NIH started requiring that investigators test their hypotheses in both biological sexes because it has become apparent that metabolism and other physical processes are very different in males vs. females [15]. Since this update to the NIH guidelines, there has been an influx of new research demonstrating that a variety of fungal infections/colonizations show sex-specific differences in a broad range of organisms, including humans and several mammalian species and insects.

Thus, the aim of this review was to examine the literature since 2016 showing different types of sex-specific patterns in fungal infections/colonizations, whether that be differences in immune responses, fungal diversity, or mycobiota composition, and determine if the mechanisms responsible for these differences have been identified.

## 2. Sex Differences in Response to Fungal Infections That Cause Disease in Mammals and Invertebrates

### 2.1. Mice

A recent paper measured the impact of airborne *Aspergillus fumigatus* (Phylum: Ascomycota) in six-week-old C57BL/6 mice to mimic human nose-only exposure and determine significant predictors of the immune response against these fungi [16]. Male and female mice were exposed to three weekly exposures of *A. fumigatus* and were then euthanized on day 3 or 28 days after the last exposure.

Female mice exhibited significantly higher antibody titers and expression fold changes. For example, as compared to male mice, bronchoalveolar lavage fluid IgG2a titers were significantly higher in naïve and *A. fumigatus*-challenged female mice, reflecting overall higher immune responses in females [17]. Additionally, while there was no difference in cytokine expression between male and female mice, the fold changes in neutrophils (70- vs. 52-fold), eosinophils (462- vs. 258-fold), and lymphocytes (80- vs. 53-fold) were significantly higher in *A. fumigatus*-challenged female mice on day 3 post-challenge, whereas only the fold change in lymphocytes (46- vs. 28-fold) was significantly higher in *A. fumigatus*-challenged female mice on day 28 post-challenge. Finally, serum IgG2a and IgE titers in *A. fumigatus*-challenged female mice were observed to be higher, but failed to reach statistical significance at *p* < 0.05. This research corroborates similar findings that show increased immune responses in females in response to other fungal pathogens [18,19].

C57BL/6 male mice also appear to show more susceptibility to systemic *C. albicans* (Phylum: Ascomycota) infection than female mice [20], as only 14% of male mice survive after intravenous infection compared to 86% survival of female mice. Interestingly, this difference in survival was not due to differences in organ fungal burden, as male and female mice had similar fungal loads. To test if sex hormones were responsible, both male and female mice were gonadectomized, treated with 5α-dihydrotestosterone (5αDHT), and then intravenously infected with *C. albicans*. Gonadectomized female mice treated with higher doses of 5αDHT were more susceptible to systemic *C. albicans* infection, as assessed by survival, and had similar survival to that of non-gonadectomized males. These data suggest that 5αDHT suppresses mouse resistance to systemic *C. albicans* infections, which is not terribly surprising, as testosterone is known to suppress the immune response [21]. Similarly, BALB/c male mice are more susceptible to infection with *P. brasiliensis* (Phylum: Ascomycota) than female mice [22] and gonadectomized female mice treated with testosterone showed increased susceptibility to infection, again suggesting that testosterone suppresses the immune response.

For *C. neoformans* (Phylum: Basidiomycota) infections, BALB/c mice also appear to show a sex-specific difference, with males having a significantly higher fungal burden in the spleen than female mice during chronic infection [19]; however, there is no difference in overall mortality between the sexes. Since this strain of mice has been shown to have a Th2 response to infection with the H99 strain of *C. neoformans* [23], potential differences in the extent of the Th2 response between males and females may explain the differences in fungal burden. However, this hypothesis needs to be tested in future studies.

These studies underscore the importance of understanding sex-specific immune responses in fungal infections and pave the way for future investigations into gender-based disparities in host defense mechanisms.

### 2.2. Drosophila

A recent review from the University of Edinburgh lays out several theories for why there are differences in immune responses between males and females in *D. melanogaster* when it comes to bacterial, viral, and fungal infections [24]. Specifically, male *Drosophila* are more likely than their female counterparts to survive infection with the most commonly studied fungal infection model, *Beauveria bassiana*, when flies are challenged via spore inoculation [25]. A similar male bias in survival is observed when flies are infected with the soil fungus *Metarhizium anisopliae* [26]. In contrast, male *Drosophila* are more susceptible to systemic challenge of *C. albicans* via intra-thoracic injection [27]. Interestingly, *Toll-1* and *Toll-7* mutant males had a greater susceptibility to infection with *C. albicans*, while *Toll-7* mutant females demonstrated resistance similar to that of controls, suggesting that male *Drosophila* exhibit a more muted immune response to *C. albicans* infection.

This paper introduced the handicap hypothesis [28], a concept suggesting that resource allocation regarding immunity and other traits related to selective advantage differs between males and females [29]. For instance, in males, the benefits of heightened mating success achieved through increased investment in sexually selected traits or costly behaviors are expected to outweigh the costs of reduced lifespan due to disease. Conversely, females are likely to prioritize investment in immunity over males to optimize their reproductive potential.

As an example of the evolution of dimorphism observed through natural selection, females lay their eggs in rotting fruits, where they are often exposed to a microbe-rich environment [24]. This in turn may lead to female immune systems that are better trained to fight off infection vs. their male counterparts, as observed by the significant investment in terms of resources.

Another hypothesis posited that the observed disparities in immune responses between males and females revolve around sexual reproduction. For instance, the absence of X chromosome transmission from fathers to their sons suggests that evolutionary processes driven by sexual selection would progress more slowly for traits predominantly influenced by the X chromosome compared to those governed by autosomes. Therefore, sexually antagonistic traits linked to the X chromosome may have a greater impact on sexual dimorphism [24].

### 2.3. Cats

A recent review from Indonesia described sex-specific responses to cases of dermatophytosis (Phylum: Ascomycota). This study identified cases of dermatophytosis in cats and showed a trend for a higher incidence in female cats than in male cats [30]. More than 90% of dermatophyte infections in cats are caused by *Microsporum canis* (*M. canis*) [31], which also causes infection in humans, and rarely in other animals, such as horses, cattle, goats, sheep, rabbits, and pigs [32]. However, sex-specific differences after infection with *M. canis* have only been observed in cats.

The increased prevalence of dermatophytosis in female cats remains unexplained. One theory suggests that stress hormones play a significant role. Chronic stress is known to modulate hormones, such as glucocorticoid cortisol, which suppresses the host immune response to fungal infections. Specifically, cortisol downregulates the proinflammatory cytokine tumor necrosis factor-α, interferon-γ, and interleukin-2, leading to a weakened immune defense against these infections [33,34]. Thus, future studies should determine if levels of these proinflammatory cytokines are decreased in female cats compared to male cats infected with *M. canis*.

### 2.4. Bats

Similarly, a recent study discussed differences in infection susceptibility in bats to *Pseudogymnoascus destructans* (*P. destructans*) (Phylum: Ascomycota) based on sex [35]. *P. destructans* causes white-nose syndrome (WNS), a condition that infects skin tissues, resulting in evaporative water loss and elevated torpid metabolic rates, ultimately leading to increased arousal frequency that results in the premature exhaustion of fat stores during hibernation [36,37]. Understanding the mechanism of WNS is crucial due to its significant impact on bat populations, as this disease is known to cause substantial mortality among bats, which has serious ecological and economic consequences. Bats play a vital role in controlling insect populations that harm crops. Without bats to naturally manage these pests, agricultural yields might decline, and farmers would need to use more pesticides, leading to increased production costs and potential environmental harm [38].

Female bats are more susceptible to disease than males after infection with *P. destructans* [35]. More female brown bats were infected and females had higher fungal loads at the beginning of winter hibernation. Additionally, females were less likely to be recaptured over winter, suggesting that females had higher mortality. The study presents several theories to why this may be the case. The first theory discusses the relationship of energy expenditure differences between male and female bats and susceptibility to infection. Differences in autumn mating behavior likely affected susceptibility to infection as females were less active than males and used torpor more extensively because they store sperm and delay ovulation until spring. Thus, this would provide more favorable conditions for pathogen growth for a longer period compared to that for males [35].

The second theory is explained by the difference observed in males and females of the maintenance of optimal temperature for fungal growth and infection. For example, females hibernate longer, which keeps their body temperature lower and in an optimal range for pathogen growth. *P. destructans* has a temperature growth range of 0–20 °C [35]. Female bats also use fats stores acquired in autumn more slowly than males, suggesting they are less euthermic (able to maintain normal body temperature) and have a more substantial constraint on energy to spend on immune responses [39,40]. Inversely, male bats may be able to inhibit pathogen growth because they are more euthermic, which allows them to mount a more robust immune response than their longer-hibernating female counterparts [41,42]. Both situations may be occurring, suggesting that other fungi might also pose a risk to hibernating bats, with resulting sex-specific patterns. These findings highlight the intricate interplay between biological factors, such as energy expenditure, hibernation patterns, and temperature regulation, and how these factors influence infection susceptibility among bats. Future research should explore whether other fungal infections display similar sex-specific patterns and examine the potential variation in infection severity.

## 3. Sex Differences in Responses to Fungal Infections That Cause Disease in Humans

### 3.1. C. neoformans

Previously, we reviewed the human sex bias in *C. neoformans*, *C. albicans*, and *P. brasiliensis* infections [12,13,14]. With both *C. neoformans* and *P. brasiliensis* infections, males are more susceptible, whereas females are more susceptible to *C. albicans* infections. For *C. neoformans*, over 70% of infected individuals are male, who are also three times more likely to be hospitalized and die from the infection compared to females. Furthermore, individuals with HIV/AIDS, irrespective of their sex, are at a heightened risk of *C. neoformans* infection [43]. Since these papers were published, there have been only a few recent studies that discuss sex-specific patterns in human fungal infections. These are discussed below.

A recent review highlighted the differences in *C. neoformans* infection susceptibility in humans between males and females. Females are better at fighting *C. neoformans* [44]. One theory postulated that this results from the differences in o-estrogen observed among the sexes. In general, o-estrogen is thought to contribute to immune upregulation, including T-cell and B-cell stimulation [45,46]. Increased phagocytosis is also observed in the presence of synthetic o-estrogen and diethylstilbestrol [19,47,48]. Additionally, estrogen has been shown to inhibit the growth of *C. neoformans*, suggesting that estrogen both upregulates the female immune response and directly affects the growth of the yeast [49,50]. These data corroborate the findings that *C. neoformans* proliferates to a higher degree in males, and lower CD3+, CD4+, and CD8+ T-cell percentages are observed in males as compared to females, suggesting that there may be an inherent deficit in T-cell responses in males [19], likely due to the immunosuppressive effects of testosterone or lower levels of estrogen in males.

A review from Slovenia also hypothesizes that for *C. neoformans*, sex hormones are likely the cause of the differences in immune responses observed between men and women [51]. Estrogen plays a protective role, whereas androgens/progesterone are more immunosuppressive [52]. The effects of sex steroid hormones are observed on adaptive cells, innate cells, cytokine secretion, and the production of high-affinity immunoglobulins, and may be involved in the mechanisms driving these sex-specific differences. In *C. albicans* infections in mice, estrogen disrupts neutrophil migration into the vagina and favors neutrophil arrest, making the vagina more susceptible to pathogens [53]. In contrast, in *P. brasiliensis* infections, where females are more resistant to infection [54], estrogen binds to an estrogen-binding protein that acts as a receptor-like molecule, binding to cytosolic proteins and preventing the transition of spores to mycelial/yeast forms to inhibit growth and infection [55]. For *C. neoformans* infections, females are again more resistant to infection, and it has been shown that estrogen inhibits the growth of the yeast [47,48,49,50,56]; however, the mechanism behind this inhibition remains unclear. It is possible that the mechanism may be similar to that shown for *P. brasiliensis*, described above. Further research is necessary to definitively explain how estrogen inhibits the growth of *C. neoformans*.

These findings may also explain why post-menopausal women are observed to have weaker immune systems [57]. Given the cyclical fluctuations in estrogen and progesterone throughout the menstrual cycle in females, future investigations should explore infection susceptibility across different phases of this cycle. In a similar manner, exploring the effect of age could help to better understand the difference in immune responses between males and females.

### 3.2. Tinea barbae and T. capitis

In an 11-year cross-sectional study [58], *Tinea barbae*, also known as tinea sycosis, disproportionately affected male patients. This finding is particularly noteworthy because *T. barbae* is relatively uncommon in developed countries, largely due to differences in lifestyle and hygiene practices. One theory for the increasing incidence of this condition, particularly among male patients, is the more widespread availability and frequent use of high-strength topical steroids [59]. Future research should investigate whether other behavioral factors, such as lifestyle and hygiene, contribute to the disproportionate prevalence of *T. barbae* in males.

In the same study [58], *T. capitis* was found to disproportionately affect female patients, 30% of whom had HIV and were immune-compromised. While *T. capitis* primarily causes disease in children, it may still be observed in adults and is a public health concern, especially in immune-compromised patients. Thus, future research should examine if other factors besides a compromised immune response affect female susceptibility to *T. capitis*.

## 4. Sex Differences in Response to Disease-Associated Disruptions in the Mycobiome

### 4.1. Humans

The human mycobiome, the fungal component of the microbiome, encompasses five primary sites: the oral cavity, intestinal tract, respiratory tract, genitourinary tract, and skin. Despite its high fungal diversity, the mycobiome constitutes only a small fraction of the total microbial community, accounting for less than 0.1% of the gastrointestinal tract microbiota [60]. Fungal microorganisms play crucial roles in metabolizing nutrients and facilitating digestion through enzyme and vitamin production [61,62].

Defining the mycobiome of a healthy individual is challenging due to significant inter-individual variability. Nevertheless, the most prevalent Phylum observed in healthy individuals is Ascomycota, comprising 48% to 99% of all present species. Other phyla, such as Basidiomycota and Mucoromycota, are present, but are considerably less abundant [60]. Common species within the mycobiome include *Saccharomyces cerevisiae* (*S. cerevisiae)* (Phylum: Ascomycota), *C. albicans*, and *Malassezia restricta* [60].

The gut–microbiota–brain axis is a bidirectional communication system involving the neuroendocrine system, vagus nerve, and inflammatory and immune systems [63]. Interestingly, dysregulation of the human microbiome reveals differences in vulnerability to psychotic disorders, such as schizophrenia (SCH). This is hypothesized to be due to the gut microbiota, since these organisms are essential for producing short-chain fatty acids and neurotransmitters, such as dopamine, norepinephrine, gamma-aminobutyric acid, glutamate, and serotonin [64,65,66], whose imbalances are often linked to SCH symptoms.

A recent study [67] aimed at understanding the mechanism behind SCH symptoms observed elevated levels of *C. albicans* (Phylum: Ascomycota) in the gut mycobiome of males with SCH compared to those of non-psychiatric controls. Additionally, elevated antibodies to *S. cerevisiae* in both males and females have been associated with food antigen hypersensitivity in individuals with SCH [68]. Notably, these fungi are commensal yeast species naturally present in the human microbiome. However, in the context of SCH, these yeast species are present in larger quantities, indicating an unhealthy microbiome. Known as dysbiosis, this is defined as having certain fungi that are normally present in a commensal state switch to a virulent pathogenic state where the homeostatic equilibrium of having a balanced composition of metabolites and energy is broken [69].

The study [67] also employed a double-blind, placebo-controlled probiotic cohort to assess whether a 14-week probiotic treatment could modulate the intestinal microbiome and correct the yeast imbalance. There was a significant reduction in *C. albicans* antibodies in males over the 14-week period, but this was not observed in females. However, males and females tend to be diagnosed with SCH in fairly equal numbers. Thus, the authors suggested that further studies are needed to explore the relationship between sex differences and the impact of probiotic treatments on the mycobiome.

This emerging evidence highlights the importance of understanding sex-specific differences in microbiome composition and function, particularly concerning fungal elements, to develop targeted therapeutic strategies for conditions like SCH.

### 4.2. Primates

Gut microbiota variations are not limited to humans. An approach to elucidate the observed disparities in immune responses between male and female primates may involve investigating the role of the microbiome and fecal matter. A study published in 2018 examined the fecal fungal composition in Tibetan macaques, revealing notable variations across sexes [70]. Specifically, female samples exhibited enrichment of two taxa, namely the family Mycosphaerellaceae and genus *Devriesia*, whereas male samples demonstrated significant enrichment of two other taxa, namely the Phylum Ascomycota and family Tetraplosphaeriaceae. Researchers were unable to definitively ascertain the underlying reasons behind these sex-specific differences. The authors postulated that such variations might stem from disparities in digestion and metabolism influenced by sex hormones. This research area holds promise for further investigation, potentially revealing important insights into how the microbiome influences the immune response.

### 4.3. Mosquitoes

In a 2020 study [71], researchers investigated the gut microbiota of adult male *Aedes aegypti* exposed to the fungal symbiont *Zancudomyces culisetae* during the larval stage. Fungal influence significantly altered the host microbiome, leading to decreased overall diversity, as measured by the Simpson and Shannon diversity indices. The fungal-exposed group exhibited a significantly higher relative abundance of the Burkholderiaceae family of bacteria, approximately 95%, compared to only 25% in the non-fungal-exposed control group. Within the fungal-exposed group, *Delftia* and *Herbaspirillum* made up 50% and 30% of the Burkholderiaceae family, respectively.

This finding contrasts with an earlier study, which observed a higher prevalence of *Burkholderiaceae* in adult female mosquitoes that did not undergo larval fungal colonization [71,72]. These results highlight sex-specific microbiome dynamics in mosquito populations influenced by the presence or absence of fungi during the larval stage, warranting further research to better understand the sex-dependent effects of fungal exposure and their implications for disease susceptibility.

A study published in 2022 [73] investigated the mycobiota of *Aedes albopictus* (Asian tiger mosquitoes) in relation to fructose metabolism among the two sexes. Similar fungal genera were present among males and females; however, the dynamics varied over time. For example, *Aureobasidium*, a genus previously identified as part of the core mycobiota of the Asian tiger mosquito, was the genus more enriched in female mosquitoes at 4 h post feeding, whereas in males, this genus was more enriched after 10 h. Additionally, levels of *Aureobasidium* in females decreased at the 10 h mark, which was marked by the increase in the mycobiota composition of *Aspergillus*, *Saccharomyces*, and *Candida* compared to other fungi. Conversely, the increase in levels of *Aureobasidium* in males 10 h post feeding was associated with a decrease in the *Candida* mycobiota composition.

This research area could potentially reveal important insights into the sex-specific differences observed in the microbiome and the impact it has on the immune function and development of an adult mosquito. Future research may explore if the variation in mycobiome activity alters the production of essential amino acids in mosquitoes, as this has been observed in insects [74].

## 5. Further Directions and Conclusions

These studies indicate that there are sex-specific differences in the response to a fungal infection or colonization in a broad variety of organisms (Table 1).

For many of these examples, females fare better. One theory postulated by Fink and Klein claims that natural selection may have a role in the consequences observed in females having better immunological protection than males [76]. For example, natural selection allows for the observance of increased antibody production in females due to the production and eventual transfer of antibodies carried through the placenta and breast milk, which is crucial for survival and reproductive success.

Finally, exploring age as an alternative avenue to comprehensively grasp the distinctions in response to fungal infections between males and females warrants further investigation. It is known that the human mycobiome is extensively shaped after birth and is influenced by factors such as delivery method, breastfeeding vs. formula feeding, and microbial colonization from relatives. As individuals mature, diet, medication, and the environment further impact the strength of the immune system. Consequently, immune responses differ significantly between children and the elderly [60].

Regarding age, a recent study delved into *Tinea capitis* occurrences within the Algerian population [75]. Among the samples analyzed, the sex ratio was determined to be 1.09 (52.2% males vs. 47.8% females) among infected patients, with children aged 4–6 years comprising the bulk (43.3%) of infections. Despite these observations, researchers were unable to draw definitive conclusions regarding the significant prevalence of infection among children in the study. This merits future exploration, as it could offer valuable insights into potential correlations between infection susceptibility, sex, and age.

A confounding factor in explaining the increased prevalence of diseases, such as *C. neoformans*, in males over females may be due to males exhibiting more risk-taking behaviors. For instance, it is known that 76% of people living with HIV, a condition that increases susceptibility to *C. neoformans* infection, are male [77]. Similarly, behavioral factors may also explain the male bias observed in fungal infections, such as *T. barbae*. For example, male patients tend to use high-strength topical steroids more frequently, which weaken the immune system and increase their susceptibility to infection [59].

**Figure 1:**
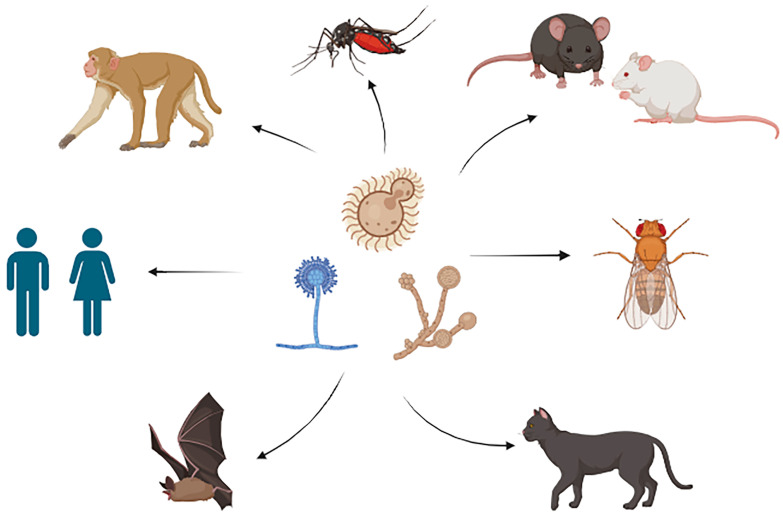
Various fungi and hosts that show a sex bias during infection or colonization. Figure created using BioRender.

Thus, much work is required to unravel the complexities of male/female host–pathogen relationships in fungal diseases. This is particularly important considering that, as of this writing, only eight years have passed since the NIH mandated that new research must include both biological sexes [15]. However, this review has revealed that sex-specific differences occur in a broad range of organisms in response to fungal infection or colonization (Figure 1), but that in many cases, the mechanisms driving these differences remain unclear. Continued exploration in these areas promises to uncover valuable insights into the mechanisms underlying sex disparities in responses to fungal infections, ultimately informing targeted interventions and therapeutic strategies.

## Figures and Tables

**Table 1:** A summary of sex-specific responses to the fungal infections/colonizations reviewed here.

Organism	Fungi	Sex-Specific Difference	*n* = Sample Size	Sex Bias Ratio *	Reference(s)
C57BL/6 Mice	*Aspergillus fumigatus*	Increased immune responses exhibited in females.	6–7 mice/ group	2:1 (F/M)	[16]
C57BL/6 Mice	*Candida albicans*	Males are more susceptible to systemic infection than females.	10 mice/ group	1:6.1 (M/F)	[20]
BALB/c Mice	*Paracoccidioides brasiliensis*	Males are more susceptible to infection than female mice. Gonadectomized female mice treated with testosterone showed increased susceptibility to infection.	3 mice/ group	2.55:1 (M/F)	[22]
BALB/c Mice	*Cryptococcus neoformans*	Males have a significantly higher fungal burden in the spleen than female mice during chronic infection.	4–5 mice/ group	9:1 (M/F)	[19]
*Drosophila*	*Beauveria bassiana*	Males are more likely to survive infection.	500–600 flies/group	1.1:1 (M/F)	[25]
*Drosophila*	*Metarhizium* *anisopliae*	Males are more likely to survive infection.	30 flies/ group	1.1:1 (M/F)	[26]
*Drosophila*	*Candida albicans*	Males are more susceptible to systemic challenge via intra-thoracic injection.	20–30 flies/ group	4:1 (M/F)	[27]
Cats	*Microsporum canis*	Females are more likely to be infected.	199 female and 74 male cats	7:1.4 (F/M)	[30]
Bats	*Pseudogymnoascus destructans*	Females are less likely to be recaptured due to death.	665 female and 1071 male bats	0.7:1 (F/M)	[37]
Humans	*Cryptococcus neoformans*	Males have lower CD3+, CD4+, and CD8+ T-cell percentages after infection.	21 males and 19 females	2.1:1 (M/F)	[56]
	*Cryptococcus neoformans*	Increased phagocytosis observed after treatment with 5 mg diethylstilbestrol.	6 males and 3 females	2:1 (M/F)	[48]
Humans	*Paracoccidioides brasiliensis*	Males are more susceptible to infection.	492 males and 92 females	5.3:1 (M/F)	[54]
Humans	*Tinea barbae*	Males are disproportionately affected in number.	6 males and 1 female	6:1 (M/F)	[58]
Humans	*Tinea capitis*	Females are disproportionately affected in number.	9 females and 6 males	1.5:1 (F/M)	[58]
Humans	*Tinea capitis*	Male children aged 4–6 years comprise 43.3% of infections.	45 males and 42 females	1.09:1 (M/F)	[75]
Human (Mycobiome)	*Candida albicans (Ca)*	Levels were elevated in the male mycobiome of those exhibiting symptoms of schizophrenia.	165 *Ca*^+^ males and 219 *Ca*^−^ males	1.3:1	[67]
Human (Mycobiome)	*Saccharomyces cerevisiae (Sc)*	Males with recent-onset schizophrenia had elevated levels of IgG antibodies to *Sc*.	51 males and 16 females	3.2:1 (M/F)	[68]
Human (Mycobiome)	*Saccharomyces cerevisiae (Sc)*	Females with non-recent-onset schizophrenia had elevated levels of IgG antibodies to *Sc*.	79 females and 114 males	1.4:1 (F/M)	[68]
Tibetan Macaques	Family: Mycosphaerellaceae; Genus: *Devriesia*	Females demonstrate significant enrichment of these taxa in the gut microbiota.	18 females and 13 males	NA	[70]
Tibetan Macaques	Phylum: Ascomycota; Family: Tetraplosphaeriaceae	Males demonstrate significant enrichment of these taxa in the gut microbiota.	18 females and 13 males	NA	[70]
*Aedes* *aegypti*	*Zancudomyces culisetae (Zc)*	Males demonstrate higher levels of the Burkholderiaceae family of bacteria in the gut microbiota than females when both sexes are exposed to this fungus during the larval stage.	12 male mosquitoes and 31 female mosquitoes	9:1	[71,72]
*Aedes* *albopictus*	*Aureobasidium genus*	This genus is more enriched in the mycobiota post feeding in females at the 4 h mark.	150 male and female mosquitoes/group	10:1 (F/M)	[73]
*Aedes* *albopictus*	*Aureobasidium genus*	This genus is more enriched in the mycobiota post feeding in males at the 10 h mark.	150 male and female mosquitoes/group	3:1 (M/F)	[73]

NA = Data not available. * The sex bias ratio was estimated based on the specific data in each manuscript.

## Data Availability

No new data were created or analyzed in this study.
